# Detecting changes in seafloor elevation in sandy coastal environments using low-cost opensource tooling

**DOI:** 10.1016/j.ohx.2024.e00532

**Published:** 2024-04-18

**Authors:** Jacob L. Vincent, Alicia M. Wilson

**Affiliations:** School of the Earth, Ocean and Environment at the University of South Carolina, 701 Sumter Street, EWS 617, Columbia, SC 29208, USA

**Keywords:** Sediment level logging, Sediment erosion, Submarine groundwater discharge

## Abstract

Knowledge of sediment erosion and deposition can be useful for a variety of engineering, marine science, and environmental applications, but collecting detailed time-series measurements of the sediment–water interface can be challenging, particularly in coastal marine environments. We developed economical and open-source sediment level loggers to record sediment–water interface time-series data with accuracy up to 1 cm. The logger is composed of a programmable Circuit-Python (or Arduino) microcontroller and “breakout boards” that attach to a specially designed printed circuit board (PCB) and an array of evenly spaced photoresistors enclosed in a robust waterproof housing. These instruments were paired with temperature sensors in a study off the coast of Charleston, SC in the South Atlantic Bight where heat was used as a tracer to detect the flow of porewater in the permeable coastal sediments. This approach requires accurate knowledge of the depth of temperature sensors relative to the sediment–water interface. In this application, improved knowledge of the elevation of the sediment–water interface elevation data from the sediment level loggers reduced average root mean squared errors in modeling submarine groundwater discharge by as much as 25 %. The sediment level loggers can be easily installed, withstand long deployment times, and provide long-term recording abilities suitable for a range of environments.

## Graphical abstract

**Figure:**
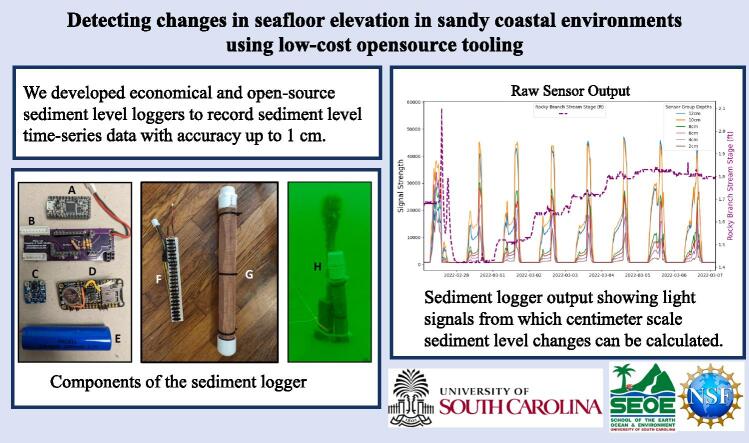

**Specifications table**

**Table:** 

Hardware name	*Sediment Level Logger*
Subject area	• Engineering and materials science • Environmental, planetary and agricultural sciences • General
Hardware type	• Measuring physical properties and in-lab sensors • Field measurements and sensors • Electrical engineering and computer science
Closest commercial analog	Lindorm Inc, SediMeter
Open-source license	*MIT LicenseCreative Commons Attribution-ShareAlike 4.0 International LicenseCERN-OHL-S v2 hardware License*
Cost of hardware	*∼$150 per unit*
Source file repository	https://10.5281/zenodo.10784048
OSHWA certification UID*(OPTIONAL)*	*OSHWA Certification #*US002132

## 1 Hardware in context

Detailed sediment transport time-series measurements can be challenging in coastal marine environments [1]. Access to remote or offshore areas is expensive and instruments must be robust enough to withstand harsh conditions for long periods with minimal maintenance and inspection. Commercial instrumentation can incur high costs that limit implementation of large scale or high-resolution data collection. The instruments described in this paper, hereafter referred to as sediment level loggers, use light sensors to detect changes in the elevation of the seafloor due to sediment loss or deposition. They are designed to be built on a low budget and provide substantial amounts of data over a long period of time with no user input after deployment.

Desirable characteristics include:

• Assembled from a small number of low-cost, readily available components
• Supports a wide variety of analog and digital sensors
• Non-proprietary software and file formats
• Removable microSD storage media
• User-adjustable operating parameters, such as sampling interval
• Operating life span > 1 year using reusable lithium or alkaline AAA batteries
• Rugged, chemically resistant environmental housing for submerged or buried deployments

Our sediment level loggers rely on inexpensive microcontrollers. These microcontrollers have become increasingly popular for both home and academic use over the past 15 years [2]. Researchers now have access to a free and expansive online community where they can learn the small amount of electrical and coding experience necessary to create their own instruments, including these microcontrollers.

**Fig. 1:**
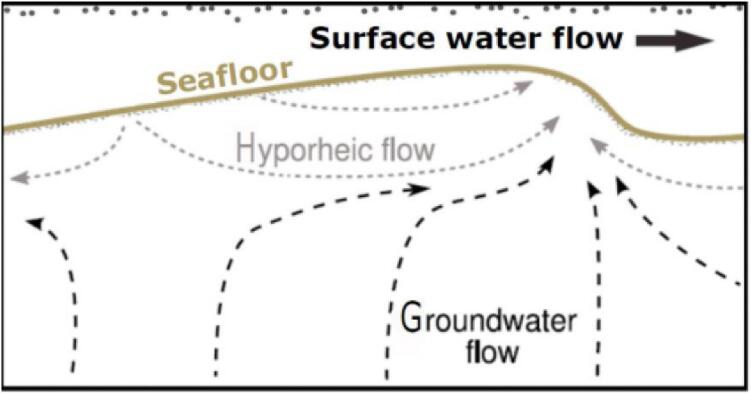
Conceptual diagram of hyporheic flow and groundwater discharge (afterBhaskar et al., 2012) [4].

The sediment level loggers in this paper were developed to support detection of hyporheic flow through benthic sediments using heat as a tracer [3]. Hyporheic flow is the transport of surface water through sediments in flow paths that return to surface water (Fig. 1). Monitoring this flow requires precise measurements of temperature logger depth in relation to the seafloor over time.

## 2 Hardware description

### 2.1 Sensors, communication, and data storage

We designed sediment level loggers using a small number of low-cost, easily accessible components. To support long deployments, the sensors were designed to include a removable microSD storage and operate on a lifespan of > 1 year on a 2000 mAh rechargeable battery.

**Fig. 2:**
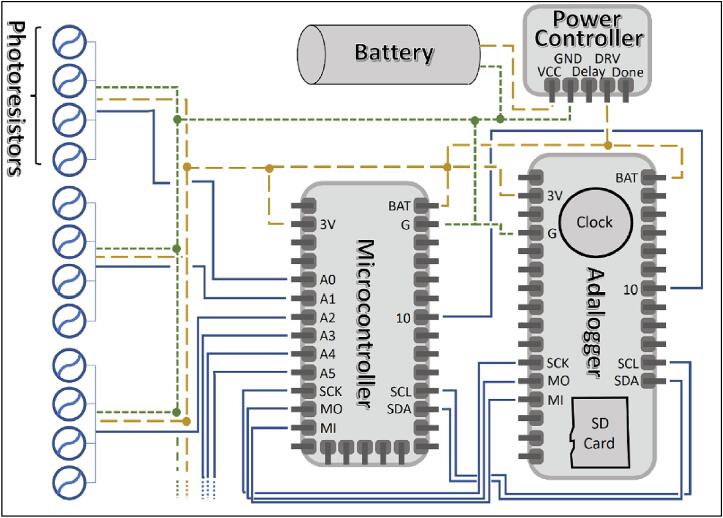
Diagram of sediment logger components.

To satisfy our goals, the sensors were made using a Circuit Python microcontroller and two breakout boards (Fig. 2). Breakout boards are additional pre-made printed circuit boards (PCBs) with sensors, chips or components that provide additional functionality to the PCB that comes with your microcontroller e.g., a clock. A PCB designed in-house was used to connect these boards to each other and the photoresistor array.

**Fig. 3:**
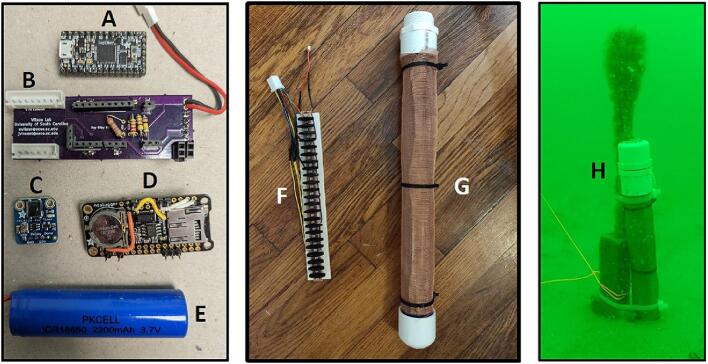
(A) Adafruit ItsyBitsy M4 Express microcontroller. (B) In-house designed PCB. (C) Adafruit TPL5110 low power timer breakout board. (D) Adafruit Adalogger Featherwing breakout board containing the PCF8523 real time clock (RTC) and MicroSD card slot. (E) Lithium Ion battery (F) photoresistor array. (G) Water resistant clear PVC housing with copper wire mesh wrap. (H) Sediment logger attached to top of temperature logging stake.

The main microcontroller of the sediment level logger is an *Adafruit* ItsyBitsy M4 Express (Fig. 3, *A*). The main processor of the microcontroller is an ATSAMD51 32-bit Cortex M4 core running at 120 MHz with 3.3 V logic with 512 KB of flash storage and 193 KB of ram. It controls a host of input and output pins, most importantly twenty-three GPIO pins and seven 10-bit analog input pins. The user should choose a microcontroller that contains enough analog inputs for their desired resolution as described in Section 2.5 of this paper.

Any microcontroller can be used to support these loggers provided that it:

• Can run Circuit Python or Arduino
• Includes enough GPIO and analog pins
• Contains enough flash storage and ram
• Fits the space requirements of the environmental housing

The PCB was created to attach all the electronics on a small circuit board in order to 1) fit into the environmental housing and 2) provide accurate connection between all of the components (Fig. 3, *B*). The *Adafruit* Adalogger FeatherWing (Fig. 3, *D*), which hosts a PCF8523 real time clock (RTC) and a microSD card slot, controls the correct logging of data. The RTC is used to keep time for the whole logging sensor. The RTC has a self-contained coin cell battery that will keep time for 5 years even when completely disconnected from the main processor. The PCF8523 in testing has been found to have an error of about 1–2 min per month, which is acceptable for our intended use. Other RTCs at higher price points are available with the same pinouts and can be used if a higher degree of timing accuracy is required. The microSD card slot allows the time series data to be recorded on a removable card for easy data retrieval when the sediment level logger is removed from the water.

The photoresistor array consists of PDV −P8001 cadmium-sulfide photoconductive (CdS) photocells spaced one centimeter apart (Fig. 3, *F*). Photocell spacing on the array is limited by the physical size of the photocells (5 mm) and width of the array supports (2 mm). Photoresistors operate by acting as a variable resistor where when they are in contact with light, the resistance decreases with more Lux. These sensors can detect light down to a minimum of 0.6 Lux which allows detection under turbid or cloudy conditions [5]. These photoresistors are commonly used in light switching mechanisms and appliances as they are very robust and inexpensive. To detect the maximum amount of light through a water column, we chose CdS cells that respond to light between 400 nm (violet) and 600 nm (orange) wavelengths, peaking at 520 nm (green), which is close to the deepest penetrating blue light (∼480 nm) [5]. This means the photocells can potentially detect light changes at their peak wavelength up to 100 m beneath the ocean surface. The array is connected to the analog to digital convertor (ADC) ports of the microcontroller via the PCB (Fig. 2). Because Photocells are a type of variable resistor, multiple can be wired together inline to a single ADC port.

### 2.2 Environmental housings

The sediment level loggers were contained in rugged water-resistant environmental housings that can be submerged for long periods of time without significant bio-fouling for easy reusability. These environmental housings were constructed of transparent clear nominal 2-inch PVC tube cut 40 cm in length (Fig. 3, *G*). The bottom of the tube was glued with a cap and the top was glued with a threaded male adapter to allow for insertion of the photoresistor array components. To prevent condensation inside the instrument, the bottom 2–3 cm of the tube was filled with a hydrating clay desiccant (cat litter) before the instruments were inserted into the housing. To prevent recording light signals that may reflect down the center of the tube, supports were added to the back of the photoresistor array so that the photocells are pressed up against the inside of the PVC tube. The top was then capped with a threaded PVC cap. Teflon tape and silicon were applied on the threads to ensure that no leaks occur. The clear part of the housing was wrapped with 0.034″ mesh sized copper wire cloth to prevent biofouling that could affect the accuracy of the light signals. Mounts made of HDPE were created to secure the sediment sensors to other instruments embedded in the seafloor. These heat-shaped HDPE strips were more robust than large zip ties and did not rust like conventional steel U-bolts. They were attached using nylon bolts and nuts (Fig. 3, *H*). We tested the durability of the wire cloth 20 m below water surface, 10 km offshore on a sandy continental shelf. The wire cloth lasted 6–8 months before it became torn, likely during the passage of two hurricanes. At the end of the deployments, the sediment sensors did not show signs of biofouling or accumulation of debris that could obstructed light from the photoresistors.

### 2.3 Software

All microcontrollers in the sediment level loggers were programmed using Circuit Python version 5 [6]. Circuit Python is based on Micropython but provides higher stability and ease of debugging.

**Fig. 4:**
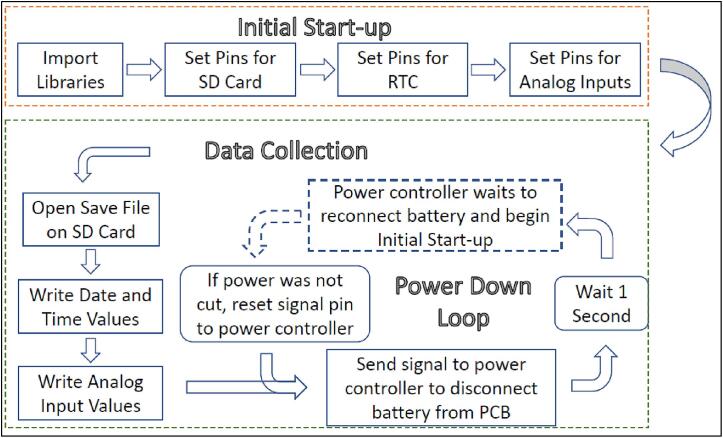
Overview of the Circuit Python code for the microcontroller. The main program follows three main phases: Initial startup, Data collection, and Power management.

The main program on our microcontroller follows three main phases: startup, data collection, and power management (Fig. 4). The startup portion of the code imports all relevant libraries needed to run the code and initializes the connections to the breakout boards and sensors connected to the microcontroller. The “Data Collection” portion of the code prepares the text file on the attached SD card for writing and then writes the current values from the clock modules and the photoresistors. The final “Power Down Loop” portion signals the power timing board to shut off power to the microcontroller and set an internal timer for two hours before re-supplying power to initiate the startup phase. The three different portions of code are necessary because the power timing board switches off power to the PCB and microcontroller between measurements erasing all the previous initialized code and data.

### 2.4 Power and runtime

The sediment level loggers rely on hardware-based power saving methods to achieve the desired battery life, instead of using software “low power” modes. To do this, we use a TPL5110 power controller breakout board (Fig. 3, *C*). The TPL5110 timer works as a remote switch, allowing battery power to pass from the battery to the PCB only at certain time intervals. When the final data has been written to the MicroSD card, the last line of the script sends a pulldown signal back to the power controller breakout board to switch off power to the PCB. The parasitic current used by the power controller when not supplying the PCB is 20 μA. When the PCB is supplied power and recording data, the total draw is only 60 μA. Our current recording interval has the PCB being supplied power for 2.5 s every two hours. With a 2200mAh battery (Fig. 3, *E*), the lifespan of the sediment level logger is calculated to be over 3 years. After a three-month test logging 20 m underwater, the voltage on the battery fell only 2 % from when it was measured during installation, consistent with the power consumption calculations.

### 2.5 Data processing

Data from the light sensor goes through two post-processing steps to calculate sediment elevation. The first step is to smooth out noise in the data caused from changes in turbidity and cloud cover without losing the daily peak signal inputs. The analog input the sensor collects takes direct measurements of voltage in a 16-bit value (0–65535). As photoresistors are exposed to greater amounts of light, their resistance decreases. As the resistance increases, the voltage decreases. This leads to more light resulting in higher voltage. In total darkness, the sensor will read 0v (0bits) and read 3.3v (65535bits) in direct unfiltered sunlight. We apply an equiripple lowpass Finite Impulse Response (FIR) filter because it preserves the recorded peak signals while smoothing out the data. The equiripple FIR filter uses coefficients generated from an N of 30 and an OmegaP and OmegaS of 0.20 and 0.25, respectively. The second step finds the sediment–water interface. This process locates the maximum value that each photoresistor group recorded each day. The maximum for each sensor is then normalized relative to the uppermost (always unburied) and lowermost (always buried) sensors to determine the location of the true sediment line. For example, if the top 6 cm (3 groups) of photoresistors all read full daylight, they will trend together with high values. Conversely, the groups of photoresistors that are fully buried trend together with low values. When a group of photoresistors is partially covered, we can accurately calculate the fraction that are covered by calculating the percent signal strength relative to the fully uncovered and buried groups. If multiple photoresistors share an ADC port, the recorded signal (voltage) is additive because the photoresistors are connected in series. The percentage of covered photoresistors in each group can be calculated with:

$$\frac{\mathrm{Recordedv}}{\begin{array}{c} (Peakvoffullyuncoveredgroup-\\ Peakvoffullycoveredgroup) \end{array}})*100=\%ofgroupuncovered$$

With *v* being the voltage signal recorded. With a pair of photoresistors, the signal strength of that group will be 50 % when one sensor is buried. This indicates that the sediment level is in between those photoresistors.

## 3 Design files summary

**Table:** 

**Design file name**	**File type**	**Open-source license**	**Location of the file**
*PCB_v2*	Gerber Files	*CERN-OHL-S v2 hardware License*	https://doi.org/10.5281/zenodo.10784048
Sensor_diagram.png	Diagram	*CERN-OHL-S v2 hardware License*	https://doi.org/10.5281/zenodo.10784048
Diagram_of_components.png	Diagram	*CERN-OHL-S v2 hardware License*	https://doi.org/10.5281/zenodo.10784048
Photoresistor_wiring.png	Diagram	*CERN-OHL-S v2 hardware License*	https://doi.org/10.5281/zenodo.10784048
tray_and_supports.stl	STL file	*CERN-OHL-S v2 hardware License*	https://doi.org/10.5281/zenodo.10784048

• The PCB folder contains all of the Gerber files necessary to create or order your own PCB for this project.
• The Sensor_diagram.png is an overview of how all the completed components are connected to one another.
• The Diagram_of_components.png illustrates the assembly of components described in the Bill of materials.
• The Photoresistor_wiring.png describes how to wire the photoresistor array based on how many photoresistors you require per analog channel.

## 4 Bill of materials summary

**Table:** 

**Designator**	**Component**	**Number**	**Cost per unit − currency**	**Total cost − currency**	**Source of materials**	**Material type**
A1	ItsyBitsy M4 express	1	$14.95	$14.95	adafruit.com	Microcontroller
A2	Short Feather Male Headers	2	$0.50	$1.00	adafruit.com	electrical components
B1	PCB Board	1	$2.00	$2.00	jlbpcb.com	PCB
B2	Short Feather female Headers	2	$1.50	$3.00	adafruit.com	electrical components
B3	*Through-Hole Resistors − 2 K ohm 5 % 1/4W*	1	$0.08	$0.08	adafruit.com	electrical components
B4	*Through-Hole Resistors − 22 K ohm 5 % 1/4W*	1	$0.08	$0.08	adafruit.com	electrical components
B5	*Through-Hole Resistors − 47 K ohm 5 % 1/4W*	1	$0.08	$0.08	adafruit.com	electrical components
B6	*Through-Hole Resistors − 100 K ohm 5 % 1/4W*	1	$0.08	$0.08	adafruit.com	electrical components
B7	6 Pin JST-PHR connector Female	1	$0.12	$0.12	digikey.com	electrical components
B8	8 Pin JST-PHR connector Female	1	$0.16	$0.16	digikey.com	electrical components
B9	2 Pin JST-PH connector Male	2	$0.10	$0.20	digikey.com	electrical components
B10	0.1″ 36-pin Strip Right-Angle Female/Socket Header	1	$0.50	$0.50	adafruit.com	electrical components
B11	0.1uF ceramic capacitor	1	$0.20	$0.20	adafruit.com	electrical components
B12	220uF 16 V Electrolytic Capacitor	1	$0.30	$0.30	adafruit.com	electrical components
C1	TPL5110	1	$4.95	$4.95	adafruit.com	Microcontroller breakout board
C2	Break-away 0.1″ 36-pin strip right-angle male header	1	$0.60	$0.60	adafruit.com	electrical components
D1	Adalogger FeatherWing	1	$8.95	$8.95	adafruit.com	Microcontroller breakout board
E1	18,650 battery w/ JST connector	1	$9.95	$9.95	adafruit.com	Battery
F1	2x8cm PCB Prototype Boards	3	$0.70	$2.10	amazon.com	electrical components
F2	Photo cell (CdS photoresistor)	24	$0.95	$22.80	adafruit.com	electrical components
F3	8 Pin JST-PH connector Male	1	$0.44	$0.44	digikey.com	electrical components
F4	6 Pin JST-PH connector Male	1	$0.34	$0.34	digikey.com	electrical components
F5	*Through-Hole Resistors − 10 K ohm 5 % 1/4W*	12	$0.08	$0.96	adafruit.com	electrical components
G1	2in SCH 40 clear PVC pipe −4ft	0.5	$51.19	$25.59	McMaster-Carr	Building Materials
G2	2in SCH 40 PVC cap	1	$2.60	$2.60	Lowes / Home improvement store	Building Materials
G3	2in SCH 40 PVC cap threaded	1	$4.54	$4.54	Lowes / Home improvement store	Building Materials
G4	2in SCH 40 PVC cap threaded adapter	1	$2.60	$2.60	Lowes / Home improvement store	Building Materials
G5	Copper Wire Cloth, 80 x 80 Mesh Size, 0.007″ Opening Size, 2 Feet x 2 Ft	1	$35.68	$35.68	McMaster-Carr	Building Materials

## 5 Build instructions

Building the instrument consists of three stages: (5.1) assembling the electronics and sensor array, (5.2) creating the Environmental housing, and (5.3) uploading the software.

### 5.1 Assembling the electronics

**Fig. 5:**
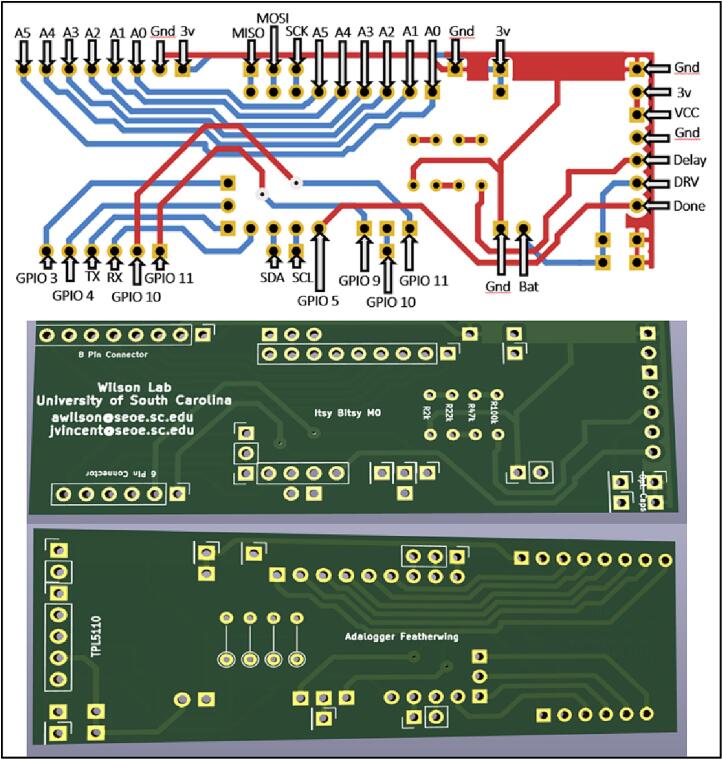
PCB (B1) routing schematic and finished board.

**Fig. 6:**
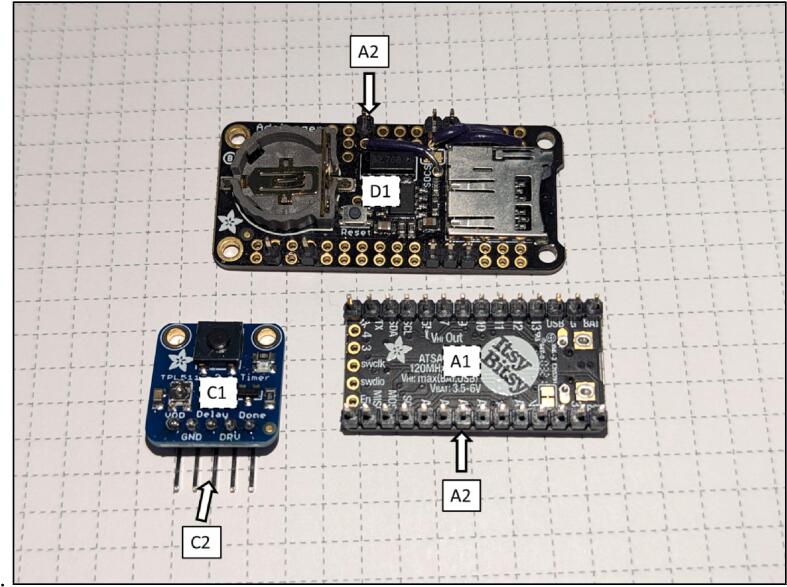
M4 Express microcontroller (A1), TPL5110 (C1), and Adalogger (D1).

**Fig. 7:**
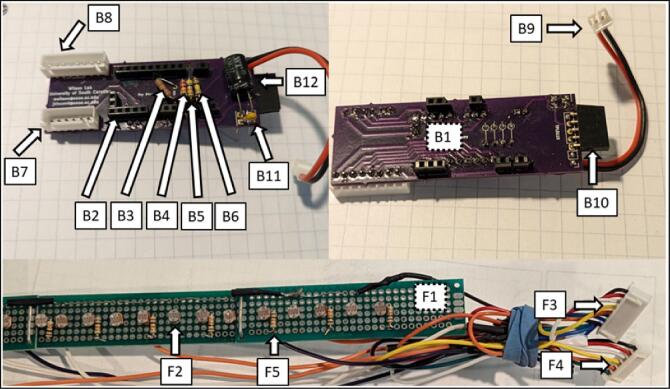
PCB board (B1) and sensor array (F1) and of components that attach to them in the *Bill of materials summary.*

Building the electronics consists of first creating or buying the PCB (B10) (Fig. 5) using the gerber files in the linked *Source File Repository* and soldering on all components (B1-B12) shown in Fig. 6, Fig. 7.

The PCB (B1) has silk screen markings showing where all the components should be soldered to (Fig. 5). Male short pins (A2) are soldered on to the Adalogger Featherwing (D1), ItsyBitsy M4 express (A1), and right-angle headers (C2) of the TPL5110 (C1). The sensor array must be made to the desired length by connecting PCB prototyping boards (F1). The user then solders the photoresistors (F2) and resistors (F5) to the prototype boards at the preferred spacing. Fig. 9 diagrams how to connect single or multiple photoresistors per analog channel. A support tray design file (tray_and_supports.stl) included in the Design file summery of this paper can be copied and created by the user to replace the prototype boards.

**Fig. 8:**
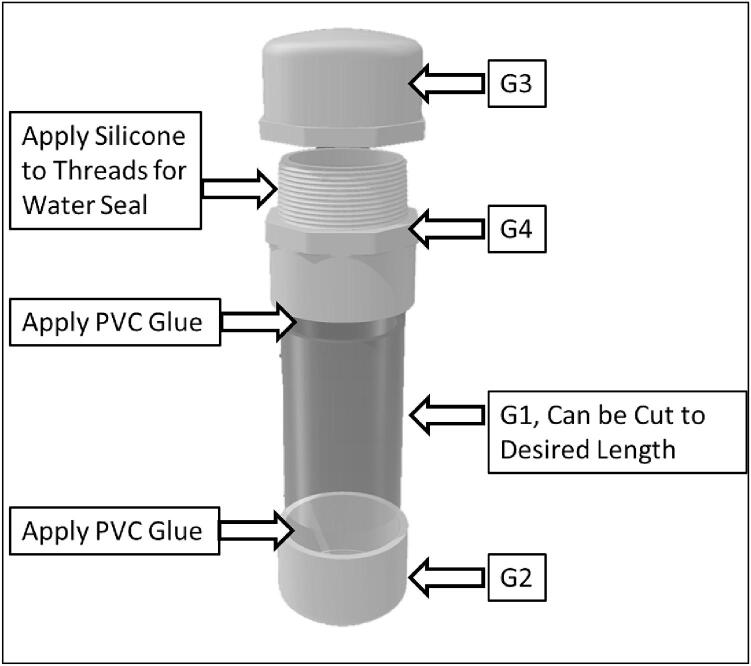
Assembly of Environmental Housing.

**Fig. 9:**
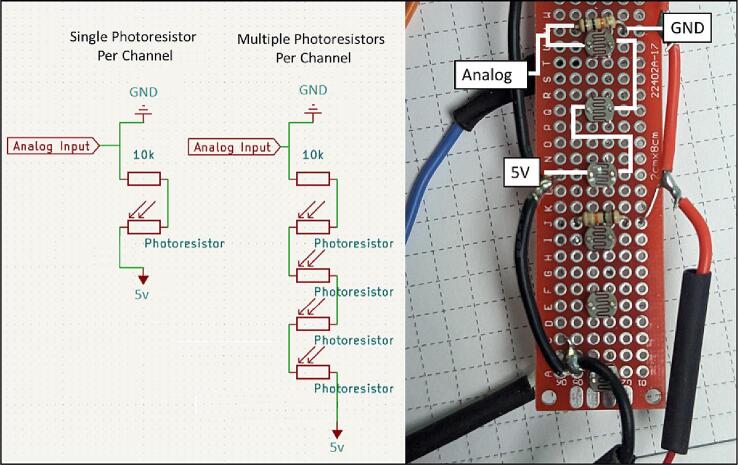
Diagram of photoresistor wiring for single or multiple photoresistors per analog channel. The photo shows the traces on the diagram overlayed a built section of the photoresistor array. The example array in the photo has 3 photoresistors per channel. Note: the ground and 5v wire colors are reversed from the customary “red = hot” and “black = ground”. (For interpretation of the references to colour in this figure legend, the reader is referred to the web version of this article.)

### 5.2 Environmental housing

The environmental housing is built by first cutting a piece of clear PVC tube (G1) to the appropriate length, which is about 15 cm longer than the desired length of the sensor array. Then the bottom cap (G2) and top male threaded adapter (G3) are glued to the ends of the PVC tube (Fig. 8). If the sediment level logger is to be used in a marine environment the copper cloth (G5) should be secured to the PVC tube to prevent biofouling and preserve sensor accuracy.

### 5.3 Uploading software

The Circuit Python bootloader must be installed onto the micro controller before the user uploads the logging script. Online instructions to install the bootloader can be found on circuitpython.com [6] for many different types of microcontroller. After the bootloader is installed, simply dragging the “main.py” code and “lib” folder supplied in the repository onto the microcontroller will install the necessary codes to run the instrument. The instrument will automatically run and record measurements to the microSD card as soon as the battery is inserted.

## 6 Operation instructions

**Fig. 10:**
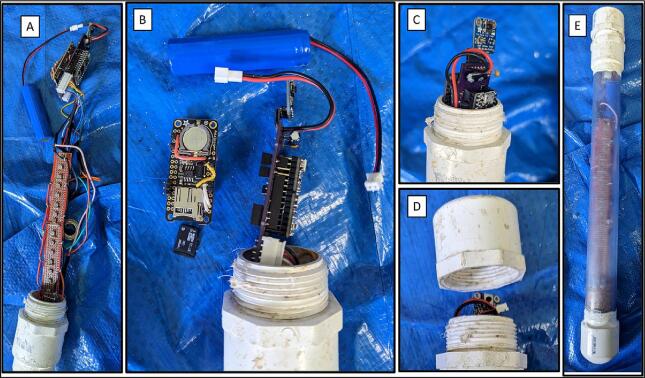
(A) Inserting array into environmental housing. (B) Battery and microSD card replacement (C) PCB orientation for insertion into environmental housing. (D) Sealing the environmental housing. (E) Full housing with array all components inserted.

Operating the sediment level loggers is straightforward. The user must first decide how they are going to affix the logger to the sediment surface. The Sediment level logger has proven to work well attached to fence stakes, wells, and other seafloor instruments that are imbedded deep enough to stay in place during high flow events. The logger has been shown to work in semi − turbid environments with visibility as low as 15–30 cm, but may not be accurate in excessively turbid environments. The user then connects the battery to the PCB and inserts the assembly into the PVC tube and screws the top cap on. Although schedule 40 PVC has a critical collapse pressure of 314PSI [7], the housing should be tested at depth before long term deployment to ensure there are no leaks. No leaks have occurred during testing up to 20 m, silicone can be applied to the PVC cap threads to ensure water does not make its way into the PVC tube under higher pressures (Fig. 8). The dried silicone is not permanent, and the user can break the seal and unthread the cap with hand tools as before. The logger automatically turns on and starts logging once the battery is connected. The instrument is then affixed to its base with either zip ties or formed HDPE strips as described in Section 2.2. Once the logger has been retrieved after deployment, the user then unthreads the PVC cap and disconnects the battery. The user can then retrieve the SD card from the Adalogger (Fig. 10).

## 7 Validation and characterization

### 7.1 Background and application

In this investigation, we used the sediment level loggers to support data modeling of groundwater flow below the seafloor. Thermal time series measurements were used to trace groundwater exchange in the upper 0–2 m of seafloor sediments. These thermal data were used to solve the inverse problem for groundwater discharge velocities and estimate the depth of hydrodynamic exchange using a program called MATTSI [8], [3].

Tracking sediment elevation changes at the centimeter scale was crucial to our current study. Testing showed that the movement of sediment by a few centimeters could cause inaccurate results in our MATTSI model. Thus, the upper portion of our thermal instruments contains a higher density of temperature loggers to track hyporheic flow. Before the introduction of sediment level loggers, we estimated the sediment depth based on temperature differences between loggers. During storms and large groundwater flushing events, predicting sediment movement using temperature loggers became difficult and inaccurate. With the introduction of our sediment level logger, we were able to approximate boundary conditions at a higher resolution (centimeter scale) and could provide estimates of hyporheic fluxes with lower errors.

### 7.2 Bench test and preliminary results

The sediment level logger was tested at the bench scale and in two deployments, one at sea and one in a freshwater stream. During both the stream and seafloor experiments, the elevation of the sediment water interface was measured during installation and before removal of the sensor to verify recordings at the start and end of deployments.

**Fig. 11:**
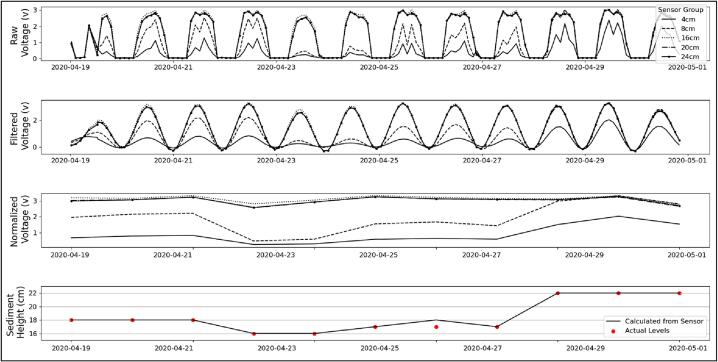
Bench test results of the sediment level logger. (A) Raw voltage collected by the photoresistors. (B) Filtered data. (C) Normalized data. (D) Calculated scour showing accretion and scour of 4 cm. Sediment depth is relative to the top of the sensor array (0 cm). Instrument was installed to 18 cm as the simulated seafloor.

The bench test (Fig. 11) consisted of installing the sensor outside in direct sunlight in a clear 3-gallon tub, then changing the sediment level every one to three days to simulate moving sediments on the bottom of the seafloor. The tub allowed light in from all sides. Pool filter sand was used as the bench test medium to represent the medium to coarse sand found throughout our field site. During the test, the logger was able to successfully track changes in sediment level to the cm scale while using 4 photoresistors per analog channel. The raw data (Fig. 8, *A*) shows that one grouping of light sensors (16 cm) failed to collect data during the bench test. This was because of a failed connection to the initial homemade PCB used. The PCB files were sent to a fabricator and professionally made mid-way through the seafloor deployments and fixed this issue.

### 7.3 Logger test 20 km offshore in sandy seabed

**Fig. 12:**
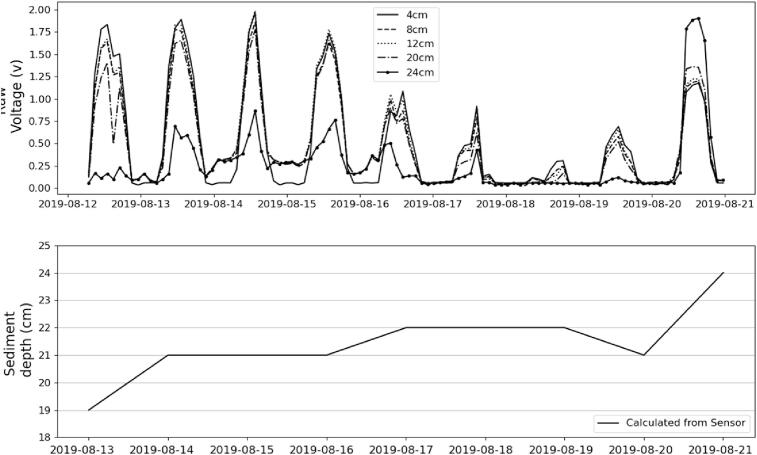
Sediment level logger results from our field site 20 km offshore of Charleston, SC. (A) Raw signal data collected by the photoresistors. (B) Calculated scour showing a large event between 8/18 and 8/20/2019 due to a hurricane. Sediment elevation is relative to top of the sensor array (0 cm). Instrument was initially installed 20 cm into the seafloor.

The loggers were tested *in situ* during 2019 (Fig. 12) at our field site, 10–20 km off the coast of Charleston SC. As before, the raw data shows photoresistor analog output caused by daylight hitting the light sensor array. The group of 4 photoresistors at the 4 cm depth (1, 2, 3, and 4 cm) did not detect light on initial deployment on 8/12/19 but were partially uncovered (about 2 of 4 light sensors) by 8/18/19. By 8/20/19, the whole array was fully uncovered when the eyewall of Hurricane Dorian approached and passed over our field site (Fig. 11). As seen in the preliminary data, the sensor tracked the sediment elevation changes before Hurricane Dorian dislodged the beta build of our sensor on 8/21/19.

### 7.4 Logger test in Rocky Branch stream

**Fig. 13:**
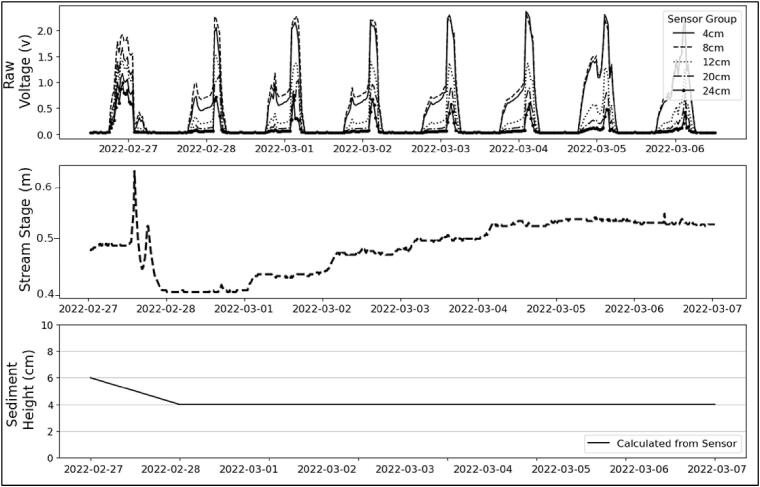
Sediment sensor raw output from Rocky Branch stream in Columbia, SC. (A) Raw signal data collected by the photoresistors. (B) Stream stage in meters. (C) Calculated scour showing 2 cm of scour after pulse in stream stage.

The logger was then installed in the Rocky Branch stream in Columbia, SC from February 24 through March 10, 2022. The timing was set to 20 min instead of 2 h as previous sensors because the deployment time was much smaller (2 weeks instead of 1 year) so battery life could be traded off for temporal resolution. The logger was initially installed so that the sediment water interface was between the second and third photoresistor (2 cm). During a small rain event on February 27th, there was a sudden spike in stream stage (Fig. 10). The change in light levels recorded scour of 2 cm, with a new sediment water interface depth of 4 cm (Fig. 13). The photoresistor array was propped away from the inside of the clear PVC tube caused by an installation error which allowed extra light reflection to hit the photoresistor array, making the initial burial conditions less clear. Nevertheless, an observable change in sediment level was still detected even though the internal structure had failed. New 3-D printed structures are being designed to replace handmade internal structures. Field observations confirmed that the housing of the sensor did not cause any measurable hydrodynamic scour downstream from where it was buried. Scour could be a concern if the instrument were to be used in areas with higher water velocities and smaller particle sizes.

Photoresistor output changes after the storm showing a clear separation of the 10 and 12 cm pairs (top of instrument) vs the rest indicating a sediment water interface between the 8 and 10 cm line. This was confirmed in the field when the sensor was removed at the end of the test.

### 7.5 Applying sediment results to our model

**Fig. 14:**
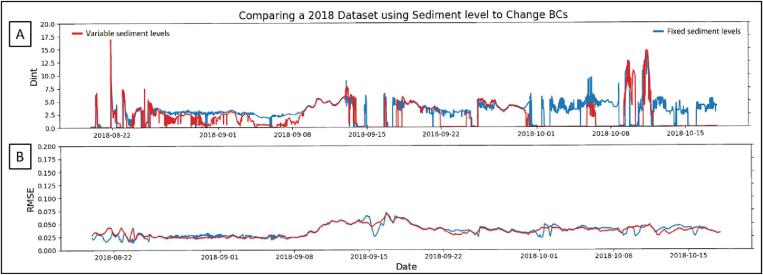
(A) Depth integrated effective dispersion (D_int_) from MATTSI model for runs with and without adjusted sediment levels based on the logger deployment. (B) Shows the root-mean-square error (RMSE) of underlying groundwater velocities from our model.

When the sediment level was applied in our heat tracer application (which models the timing and depth of hydrodynamic exchange and the underlying groundwater flow velocities), model outputs were able to calculate groundwater velocity with low root-mean-square errors (RMSE) without increasing the dispersion and depth of hydrodynamic exchange (Fig. 11). Our model, MATTSI, uses D_int_ to show increases in thermal dispersion parameters to simulate the 3-D process of heat transport in 1-D. Adjusting the domain of our model through time to match sediment levels caused a decrease of 1.1 % in the RMSE and a 39 % decrease in D_int_ (Fig. 14, *B*). The changes in sediment level have little effect on the calculated groundwater velocities. With the correct sediment level inputs, we were able to demonstrate that the model has the capacity to estimate the timing, depth and magnitude of hydrodynamic exchange and the groundwater velocity with an RMSE as low as the precision of the temperature loggers.

### 7.6 Conclusions

The sediment level logger has promising applications where the precise elevation of the sediment water interface needs to be known including studies of sediment erosion and deposition. The logger has gone through 3 years of deployments and has shown to be robust enough to handle challenging marine and fluvial conditions while still being able to record centimeter scale changes in the sediment water interface.

Tracking sediment transport is important for engineering studies such as beach renourishment projects. A recent study by Park et al. [9] was able to calculate sediment transport and volume changes using annual side scan sonar images through the Beach Erosion Research and Monitoring (BERM) program. Time series data can offer significant improvement on sonar which cannot capture major sediment transport events that happen in between the yearly cruises such as hurricanes and major storms. As the number of major storms are predicted to increase with a rise in global climate change, detailed time series of sediment movement will be useful. The amount of sediment movement that occur in a field site during these large drivers of erosion can help engineers model predicted beach health over time.

### CRediT authorship contribution statement

**Jacob L. Vincent:** Writing – original draft, Software, Methodology, Investigation, Formal analysis, Data curation, Conceptualization. **Alicia M. Wilson:** Writing – review & editing, Supervision, Project administration, Funding acquisition.

## Declaration of competing interest

The authors declare that they have no known competing financial interests or personal relationships that could have appeared to influence the work reported in this paper.
